# Review of Questions Concerning Clinical Drug Interactions in ADHD Treatment From Physicians in Norway

**DOI:** 10.3389/fphar.2020.607915

**Published:** 2020-12-18

**Authors:** Jan Schjøtt, Kristine Heitmann, Tina Bakkebø, Jan Anker Jahnsen

**Affiliations:** ^1^ Regional Medicines Information and Pharmacovigilance Centres (RELIS Vest), Haukeland University Hospital, Bergen, Norway; ^2^ Department of Clinical Biochemistry and Pharmacology, Haukeland University Hospital, Bergen, Norway; ^3^ Department of Clinical Science, Faculty of Medicine and Dentistry, University of Bergen, Bergen, Norway

**Keywords:** attention-deficit hyperactivity disorder, interactions, physician, clinical, drug information centers

## Abstract

Pharmacological treatment of attention deficit hyperactivity disorder (ADHD) is challenging due to a wide age span among patients, risk of reduced adherence, and comorbidities like psychiatric disorders and drug addiction. Drugs used for ADHD are associated with risk of interactions and adverse drug reactions due to their potent pharmacological effect. In this brief report we aimed to describe real-world problem areas concerning interactions in pharmacotherapy of ADHD. We reviewed questions to a Norwegian drug information center from physicians concerning drug-drug interactions involving ADHD drugs in the last 10-year period. Questions were retrieved by a combination of indexed and Boolean database searches, in addition to manual inspection. ADHD drugs and interacting drugs were defined according to the Anatomical Therapeutic Chemical (ATC) classification system. Interactions were classified by use of Stockley’s Interactions Checker (SIC). Answers were examined with regard to whether the advice from the drug information center was more restrictive, similar or more liberal than SIC when assessing drug combinations. We retrieved 61 questions that included assessment of 96 drug combinations, and found 33 potential interactions according to SIC. Methylphenidate was involved in more than 50% of the interactions, and interacting drugs were in nearly 70% of the cases from ATC-group N (Nervous system) with antidepressants most frequently involved. Seventy percent of the interactions were pharmacodynamic, and interactions were frequently described as potentially severe although they were based on theoretical evidence. All the 33 interactions could be handled with monitoring or adjusting dose or with informative measures, and none was contraindicated according to SIC. More than 90% of the questions came from physicians in hospitals or outpatient specialist practice, and questions mainly concerned adults. In 75% of the drug combinations that involved ADHD drugs, we found similar advice from SIC and the drug information center. Our results suggest that future drug information efforts in ADHD treatment to clinicians, including specialists in the field, should focus on psychotropic interactions.

## Introduction

Attention-deficit hyperactivity disorder (ADHD) is recognized as the most common behavioral disorder among children (Pastor et al., 2015; Thomas et al., 2015; Mahone and Denckla, 2017).

ADHD is diagnosed during childhood or adolescence, but symptoms can still be present in adults (Franke et al., 2018). The worldwide prevalence of ADHD is estimated to be 5.9–7.1% in children and 5.0% in adults (Willcutt, 2012). Children and adults with ADHD have frequently psychiatric comorbidity (Kraut et al., 2013; Sikirica et al., 2013; Katzman et al., 2017; Mac Avin et al., 2020). In children oppositional defiant disorder and conduct disorder are the most prevalent comorbid conditions. Substance use disorders become a problem during adolescence and even more so in adulthood. In adults, mood, anxiety and personality disorders as well as somatic diseases are included in the comorbidity pattern (Franke et al., 2018).

Pharmacological treatment is recommended for controlling ADHD symptoms (Banaschewski et al., 2018; Canadian ADHD Resource Alliance, 2018; National Institute for Health and Care Excellence, 2018). Drugs used for the treatment of ADHD are classified as psychostimulant drugs (methylphenidate and amphetamine derivatives) and nonpsychostimulant drugs (atomoxetine, guanfacine, clonidine). Pharmacological treatment of ADHD is challenging due a wide age span among the patients, risk of reduced adherence, and comorbidity. Pharmacoepidemiological data shows a trend favoring polypharmacy for the treatment of ADHD (Wu et al., 2018). A current concern is that the existing literature on interactions of ADHD drugs is limited (Schoretsanitis et al., 2019).

In this brief study, we aimed to describe real-world problem areas concerning interactions in pharmacological treatment of ADHD by reviewing questions to a Norwegian drug information center. We aimed to determine if questions concerned children or adults, as well as workplace of the physician. Furthermore, we described type of drugs, type of interactions, and classification of action, documentation and severity of the drug combinations. We also wanted to investigate whether our answers provided more restrictive, similar or more liberal advice than a recommended drug interaction database. A motivation for the study was to identify areas of problem to be targeted in future drug information efforts toward clinicians.

## Methods

### Material

Regional Medicines Information and Pharmacovigilance Centers (RELIS) is a Norwegian network of drug information centers providing decision support to health care professionals (e.g., physicians, pharmacists, nurses) in four health regions. The centers are associated with clinical pharmacology units in regional university hospitals, and the staff includes pharmacists and physicians with expertize in searching and critical appraisal of literature (Schjøtt, 2017). RELIS store indexed question-answer pairs (Q/As) in a full-text, searchable database (Schjøtt et al., 2012). The Q/As are indexed with occupation (e.g., physician, pharmacist) and workplace (e.g., general practice, hospital) for each inquirer. The database contains a simple search function where a drug (e.g., methylphenidate), or category (e.g., interactions) or an individual RELIS center (e.g., RELIS Vest) is entered. Simple searches can be combined with Boolean operators (AND/OR/NOT) in the database. Questions to RELIS are short clinical narratives that explain the clinical background for a question, and physicians often ask for assessment of several drug combinations in a question. We often observe that different physicians ask about the same drug combinations within a pharmacotherapy area.

### Search

**FIGURE 1:**
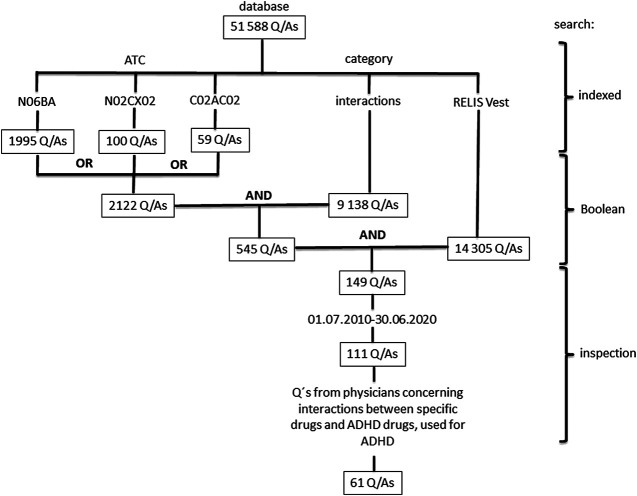
Search for questions concerning ADHD drugs and interactions in the database of the Regional Medicines Information and Pharmacovigilance Centers (RELIS) in Norway. Drugs were classified according to the Anatomical Therapeutic Chemical (ATC) classification system (WHO, 2016), and included N06BA01 (amphetamine), N06BA02 (dexamphetamine), N06BA04 (methylphenidate), N06BA09 (atomoxetine), N06BA12 (lisdexamphetamine), C02AC02 (guanfacine), and N02CX02 (clonidine). Notice that ATC-codes and generic names, categories (e.g., interactions, adverse effects) and a particular RELIS center (RELIS Vest) are indexed and searchable in the database that also include Boolean operators (e.g., AND, OR, NOT).

We performed a search of Q/As that involved drugs for ADHD. Drugs were classified according to the Anatomical Therapeutic Chemical (ATC) classification system (WHO, 2016), and included N06BA01 (amphetamine), N06BA02 (dexamphetamine), N06BA04 (methylphenidate), N06BA09 (atomoxetine), N06BA12 (lisdexamphetamine), C02AC02 (guanfacine), and N02CX02 (clonidine). The ATC-codes are searchable in the RELIS database. Notice that clonidine has an additional ATC-code; C02AC01 (C02 Antihypertensives). The search was further limited to questions posed by physicians, indexed with the category interactions, and questions received by one regional drug information center (RELIS Vest). Due to privacy issues, the questions can be slightly edited before they are stored and made public in the RELIS database. The restriction to RELIS Vest gave us the possibility to examine the exact words of the original question in our local archive. Q/As from a 10-year period (01.07.2010–30.06.2020) involving specific drugs (not drug groups) associated with treatment of ADHD and various comorbidity were included. The search strategy is shown in Figure 1.

### Protocol

The material from the search strategy was subjected to a pilot where two of the authors (JS, KH) randomized (www.randomizer.org) 10 Q/As for preliminary inspection and classification, and compared the results. The pilot Q/As were subsequently included in the study material, and a final protocol that involved all four authors was developed.

We described the unique question number to RELIS Vest, the year the question was received, age group of an individual patient (younger or older than 18 years, or unknown age), workplace of the inquirer (hospital or outpatient specialist practice, general practice or other), the ADHD drug or drugs (see above) in the question, and potential interacting drugs (according to the ATC-system). Due to expectations of a limited material, we did not perform more elaborate comparisons, and data was summarized with knowledge that it involved repetitions of drugs, drug combinations and interactions.

Interactions were classified and ranked with Stockley’s Interactions Checker (Stockley’s Interaction Checker, 2020). Stockley’s Interactions Checker (SIC) provides consistent albeit briefer information on drug interactions compared to Stockley’s Drug Interactions, and describes classification of the clinical relevance of a drug interaction. A clinically relevant interaction in SIC is classified with the following three categories: recommended action, severity, and documentation. In SIC, the recommended action for a clinically relevant interaction is either “informative”, “monitor”, “adjust dose”, or “avoid”. Recommended action for a clinically relevant interaction in the present study was defined by collapsing “monitor” and “adjust dose” into the following three levels (from low to high); “informative”, “monitor or adjust dose”, or “avoid”. In the present study, severity was classified (from low to high) as “mild”, “moderate” or “high” in concordance with SIC. Documentation in SIC is classified with four levels; “theoretical”, “case”, “study” or “extensive”. “Extensive” is an option for documentation of interactions where the information provided is based on numerous small or medium size studies or several large studies usually supported by case reports (Stockley’s Interaction Checker, 2020). Documentation of a clinically relevant interaction in the present study was defined by collapsing “study” and “extensive” to acquire the following levels (from low to high); “theoretical”, “case”, or “study”. The first ranked interaction for each drug pair was defined by the following hierarchy of categories and levels; recommended action (avoid > monitor or adjust dose > informative) > documentation (study > case > theoretical) > severity (severe > moderate > mild). The order of the categories was based on our experience that many interactions may be described as potentially severe, but recommended action (e.g., contraindicated) and documentation (study) is of more importance when providing drug information. Each interaction was defined as either pharmacodynamic or pharmacokinetic based on the description in SIC. Only one interaction was chosen from each drug pair although several interactions can be mentioned in SIC. Thus, if a pharmacokinetic interaction contained an additional description of a pharmacodynamic interaction that could not be explained by a change in plasma level of one or both drugs, the interaction was defined as pharmacokinetic.

The number of ADHD drugs, the number of drug combinations assessed and the number of interactions found in SIC were summarized. Answers were also examined with regard to whether the advice from RELIS Vest concerning the interactions included a different advice (more restrictive, similar, more liberal or not definable) than SIC.

### Statistics

Descriptive statistics was performed with SPSS version 26 (IBM Corp, Armonk, NY).

## Results

The RELIS database contained 51 588 Q/As at the end of June 2020, and RELIS Vest at this point of time had answered and indexed 14 305 of these. One hundred and forty-nine of the Q/As to RELIS Vest were associated with ADHD drugs, and 111 (74%) were from the last ten years (July 1, 2010–June 30, 2020). The number of Q/As included according to the protocol was 61 (Figure 1).

Table 1 shows characteristics of the questions that were received in the last 10-year period. The majority of the 61 questions concerned patients older than 18 years, and came from physicians in hospital or outpatient specialist practice. Only five questions came from general practice. Table 2 shows that the 61 questions concerned assessment of 96 drug combinations that included one or two ADHD drugs, with methylphenidate most frequently involved. The 96 combinations assessed involved 96 potentially interacting drugs (several drugs repeatedly assessed), and nearly 70% of the interacting drugs belonged to ATC group N (Nervous system). The most frequent enquired interacting drugs were bupropion and topiramate, which were assessed five times. Within group N, antidepressants was the most frequently assessed subgroup of drugs. Five out of eight drugs assessed three times or more were antidepressants. Three questions included assessment of combinations where two ADHD drugs were involved.

**TABLE 1:** Characteristics of questions concerning ADHD drug interactions.

Questions		*n* (%) *N* = 61
Year of question		
2010	1 (2)		
2011	2 (3)		
2012	8 (13)		
2013	6 (10)		
2014	9 (15)		
2015	6 (10)		
2016	3 (5)		
2017	7 (12)		
2018	9 (15)		
2019	6 (10)		
2020	4 (7)		
Age group of patients		
<18 years	19 (30)		
>18 years	31 (51)		
Unknown age	11 (18)		
Physicians workplace		
Hospital or outpatient specialist practice	56 (92)		
General practice	5 (8)		

Notice that percentages are rounded to whole numbers, so the sum can be different from 100. The data was collected from questions by physicians to the Regional Medicines Information and Pharmacovigilance Center (RELIS Vest), Norway.

**TABLE 2:** Characteristics of drugs in questions concerning ADHD drug interactions.

Questions	*n* (%) *N* = 61
Drug combinations assessed	*N* = 96
ADHD drugs in combinations assessed	
Methylphenidate	52 (54)
Atomoxetine	20 (21)
Lisdexamphetamine	8 (8)
Guanfacine	8 (8)
Dexamphetamine	7 (7)
Amphetamine	1 (1)
Interacting drugs in combinations assessed	
N (Nervous system)	64 (67)
A (Alimentary tract and metabolism)	6 (6)
C (Cardiovascular system)	5 (5)
D (Dermatologicals)	4 (4)
L (Antineoplastic and immunomodulating agents)	4 (4)
H (Systemic hormonal preparations, excluding sex hormones and insulins)	3 (3)
J (Antiinfectives for systemic use)	3 (3)
R (Respiratory system)	3 (3)
G (Genito-urinary system and sex hormones)	2 (2)
B (Blood and blood forming organs)	1 (1)
M (Musculo-skeletal system)	1 (1)
Interacting drugs most frequently assessed (≥3 times)	
Bupropion	5 (5)
Topimarate	5 (5)
Risperidone	4 (4)
Venlafaxine	4 (4)
Tramadol	3 (3)
Fluoxetine	3 (3)
Sertraline	3 (3)
Amitriptyline	3 (3)

Notice that percentages are rounded to whole numbers, so the sum can be different from 100. Drugs were classified according to the Anatomical Therapeutic Chemical (ATC) classification system (WHO, 2016). The data was collected from questions by physicians to the Regional Medicines Information and Pharmacovigilance Center (RELIS Vest), Norway.

Table 3 shows that 33 interactions were found (according to SIC) among assessment of 96 drug combinations found in the material. Seventy percent of the interactions were pharmacodynamic. Monitor or adjust dose was the most frequent action, documentation was mostly theoretical, but the majority of interactions were described as potentially severe. In 70 of 96 (75%) drug combinations that involved one or two ADHD drugs, we found similar advice from SIC and RELIS Vest. In 13 drug combinations (14%) RELIS Vest was more restrictive, and in 11 drug combinations (12%) more liberal than SIC. If drug combinations that gave no interaction in SIC were selected, we found 50 of 63 (79%) drug combinations with similar advice.

**TABLE 3:** Characteristics of interactions in questions concerning ADHD drug interactions.

Questions	*n* (%) *N* = 61
Drug combinations assessed	*N* = 96
Interactions	*N* = 33
Type	
Pharmacodynamic	23 (70)
Pharmacokinetic	10 (30)
Action	
Avoid	0 (0)
Monitor or adjust dose	18 (55)
Informative	15 (46)
Documentation	
Study	1 (3)
Case	11 (33)
Theoretical	21 (64)
Severity	
High	23 (70)
Moderate	9 (27)
Mild	1 (3)

Notice that percentages are rounded to whole numbers, so the sum can be different from 100. Interactions were classified and ranked according to Stockley’s Interactions Checker (Stockley’s Interaction Checker, 2020). The data was collected from questions by physicians to the Regional Medicines Information and Pharmacovigilance Center (RELIS Vest), Norway.

## Discussion

This review of real world questions concerning clinical drug interactions in ADHD treatment from physicians in Norway, showed an association of need of decision support with prescribing of combinations of psychotropic drugs. A majority of the questions came from specialists in the field, and often concerned treatment of adults with ADHD.

A majority of the potential interactions with ADHD drugs in this study was classified as potentially severe in SIC, but the interactions were usually based on theoretical evidence. Two of the five criteria identified by an expert panel assessing important interactions were the evidence and clinical implications or management burden, defined as the course of action a clinician may have to take for each potential interaction (Phansalkar et al., 2013). Thus, documentation and handling are important elements in assessment of interactions. Only seven (12%) of 58 psychotropic drug interactions had evidence from studies with a sample size of more than 100 patients according to a recent study (Nguyen et al., 2020). The possibility to detect consequences of an interaction is important as it relates potential risk of adverse effects to individual patients. Integration of drug interaction databases in dispensing software and computerized clinical decision support systems is prevalent today (Kongsholm et al., 2015). However, the interaction analysis is usually categorical, and the immediate presentation of action and severity in the interaction analysis can be potentially misleading when the description is based on theoretical evidence.

The majority of the interacting drugs in this study involved drugs from ATC-group N (drugs that affect the nervous system). This finding was not surprising since psychotropics are frequently used by patients with ADHD due to comorbidity (Kraut et al., 2013; Sikirica et al., 2013; Mac Avin et al., 2020). However, increasing psychotropic polypharmacy among children and adolescents with ADHD has been reported (Winterstein et al., 2017; Wu et al., 2018). A retrospective cohort study found that nearly 40% of children enrolled in Kentucky Medicaid, US, were exposed to psychotropic polypharmacy (Lohr et al., 2018). The most frequent psychotropic drugs in question in the present study were antidepressants. Other authors have observed that co-prescription of stimulants and antidepressants represents the most frequent therapeutic regimen in patients with ADHD (Winterstein et al., 2017).

Psychotropic polypharmacy is a clinical challenge when the current literature on interactions of ADHD drugs is limited (Schoretsanitis et al., 2019). Our questions showed that drug combinations often involve potential interactions that are pharmacodynamic and based on theoretical evidence. Compared to pharmacokinetic interactions, which can be monitored and dose adjusted, pharmacodynamic interactions often lack specific advice concerning stratification to age, gender and dose (Schjøtt et al., 2020). Furthermore, lack of consensus among databases with regard to evidence and handling of drug interactions is common (Schjøtt et al., 2020).

We found several cases of different advice between RELIS and SIC when assessing drug combinations irrespective if classified as an interaction or not. This comparison is of course limited due to the fact that SIC is used as a source when interactions are assessed by RELIS. However, RELIS always consult several sources and use our expertize to provide decision support with regard to interactions. An illustrating example was lack of any interaction between rituximab and methylphenidate or lisdexamphetamine in SIC, where RELIS mentioned implications of immunotherapy combined with ADHD treatment with risk of infections and risk of downregulation of drug metabolizing enzymes. Another example was a question where SIC found interaction between methylphenidate and nortriptyline (pharmacokinetic) and interaction between atomoxetine and nortriptyline (pharmacodynamic) where RELIS found that when nortriptyline is used for treatment of pain in a low dose the respective combinations can be used.

Questions to RELIS are spontaneous, and do not necessarily represent drug problems perceived by the general population of health care professionals. However, a majority of the questions came from experienced specialists in ADHD treatment, and frequently concerned individual adult patients. Adult patients with ADHD are expected to have more comorbidity than children, with increased risk of polypharmacy. A multinational study from five Nordic countries showed that co-medication with other psychotropics were common among adults and increased with age. Adults now constitute about half of the individuals using ADHD drugs in the Nordic countries according to prescription register data, and methylphenidate is the preferred ADHD drug (Karlstad et al., 2016). However, the diagnosis of ADHD among adults is controversial (Shah and Morton, 2013), and the long-time safety and efficacy of ADHD drugs are insufficiently studied in adults (Volkow and Swanson, 2013). Treatment of patients with ADHD irrespective of age can be particularly challenging with clinicians ending up prescribing multiple medications with risk of interactions (Childress and Sallee, 2014). If treatment of ADHD is ineffective and remaining symptoms are treated with additional drugs that induce drug interactions, this might be a reason for concern.

The material in this study is naturalistic and descriptive with risk of biased interpretation. Notably, the interaction analysis in SIC was performed in 2020 although the questions to the drug information center is from a 10-year period. It is difficult to perform meaningful comparisons due to the small size (e.g., more frequent questions about methylphenidate and bupropion concerning adults vs children with ADHD?). We are only informed about the drugs mentioned in the questions, but the patients could be using even more. We also lack complete clinical background with description of comorbidity. Interactions between other drugs than for ADHD were found, but are not described here. However, the possibility to process unstructured textual data, from for example suspected adverse drug reaction (ADR) reports, medical literature, electronic health records, and social media, is of current interest in pharmacovigilance (Ventola, 2018). Our results could be complementary to more systematic retrospective research materials, and they can be used to formulate hypotheses to be tested prospectively. Questions reflect perceived problems from health care professionals that could not be solved by other drug information sources (e.g., monographs, colleagues), and differ in this respect to ADR reports based on suspicion. Thus, we believe that it is possible to propose targets for regional and national drug information efforts from our data.

We conclude that future drug information efforts in ADHD treatment should focus specifically on psychotropic interactions with a motivation to reduce polypharmacy.

## Data Availability Statement

The raw data supporting the conclusions of this article will be made available by the authors, without undue reservation.

## Ethics Statement

Ethical review and approval was not required for the study on human participants in accordance with the local legislation and institutional requirements. Written informed consent from the participants’ legal guardian/next of kin was not required to participate in this study in accordance with the national legislation and the institutional requirements.

## Author Contributions

JS and KH designed the study. JS collected the data. JS and KH performed the pilot. All authors contributed to the final protocol and interpretation of the data. JS wrote the manuscript and all authors read and approved the final version of the manuscript.

## Conflict of Interest

The authors declare that the research was conducted in the absence of any commercial or financial relationships that could be construed as a potential conflict of interest.

## References

[B1] Banaschewski T. Coghill D. Zuddas A. (2018). Oxford textbook of attention deficit hyperactivity disorder. Oxford, United Kingdom: Oxford University Press.

[B2] Canadian ADHD Resource Alliance (2018). Canadian ADHD practice guidelines. 4th Edn Toronto, ON: CADDRA.

[B3] Childress A. C. Sallee F. R. (2014). Attention-deficit/hyperactivity disorder with inadequate response to stimulants: approaches to management. CNS Drugs 28 (2), 121–129. 10.1007/s40263-013-0130-6 24402970

[B4] Franke B. Michelini G. Asherson P. Banaschewski T. Bilbow A. Buitelaar J. K. (2018). Live fast, die young? A review on the developmental trajectories of ADHD across the lifespan. Eur. Neuropsychopharmacol. 28 (10), 1059–1088. 10.1016/j.euroneuro.2018.08.001 30195575 PMC6379245

[B5] Karlstad Ø. Zoëga H. Furu K. Bahmanyar S. Martikainen J. Kiler H. (2016). Use of drugs for ADHD among adults—a multinational study among 15.8 million adults in the Nordic countries. Eur. J. Clin. Pharmacol. 72 (12), 1507–1514. 10.1007/s00228-016-2125-y 27586399 PMC5110707

[B6] Katzman M. A. Bilkey T. S. Chokka P. R. Fallu A. Klassen L. J. (2017). Adult ADHD and comorbid disorders: clinical implications of a dimensional approach. BMC Psychiatr. 17, 302 10.1186/s12888-017-1463-3 PMC5567978 28830387

[B7] Kongsholm G. G. Nielsen A. K. Damkier P. (2015). Drug interaction databases in medical literature: transparency of ownership, funding, classification algorithms, level of documentation, and staff qualifications. A systematic review. Eur. J. Clin. Pharmacol. 71 (11), 1397–1402. 10.1007/s00228-015-1943-7 26369536

[B8] Kraut A. A. Lagner I. Lindemann C. Banaschewski T. Petermann U. Petermann F. (2013). Comorbidities in ADHD children treated with methylphenidate: a database study. BMC Psychiatr. 13, 11 10.1186/1471-244X-13-11 PMC3544568 23294623

[B9] Lohr W. D. Creel L. Feygin Y. Stevenson M. Smith M. J. Myers J. (2018). Psychotropic polypharmacy among children and youth receiving Medicaid, 2012-2015. J. Manag. Care Spec. Pharm. 24 (8), 736–744. 10.18553/jmcp.2018.24.8.736 30058983 PMC10397940

[B10] Mac Avin M. J. Teeling M. Bennett K. E. (2020). Trends in attention-deficit and hyperactivity disorder (ADHD) medications among children and young adults in Ireland: a repeated cross-sectional study from 2005 to 2015. BMJ Open 10, e035716 10.1136/bmjopen-2019-035716 PMC7204927 32327478

[B11] Mahone E. M. Denckla M. B. (2017). Attention-deficit/hyperactivity disorder: a historical neuropsychological perspective. J. Int. Neuropsychol. Soc. 23 (9-10), 916–929. 10.1017/S1355617717000807 29198277 PMC5724393

[B12] National Institute for Health and Care Excellence (2018). Attention deficit hyperactivity disorder: diagnosis and management, NICE guideline. Available at: https://www.nice.org.uk/guidance/ng87 (Accessed December 1, 2018). 29634174

[B13] Nguyen T. Liu X. Abuhashem W. Bussing R. Winterstein A. G. (2020). Quality of evidence supporting major psychotropic drug‐drug interaction warnings: a systematic literature review. Pharmacotherapy 40 (5), 455–468. 10.1002/phar.2382 32107798

[B14] Pastor P. N. Reuben C. A. Duran C. R. Hawkins L. D. (2015). Association between diagnosed ADHD and selected characteristics among children aged 4–17 years: United States, 2011–2013. NCHS Data Brief 201, 1–8. 25974000

[B15] Phansalkar S. Desai A. Choksi A. Yoshida E. Doole J. Czochanski M. (2013). Criteria for assessing high‐priority drug‐drug interactions for clinical decision support in electronic health records. BMC Med. Inf. Decis. Making 13 (1), 65 10.1186/1472-6947-13-65 PMC3706355 23763856

[B16] Schjøtt J. (2017). Benefits of a national network of drug information centres: RELIS. Eur. J. Clin. Pharmacol. 73 (1), 125–126. 10.1007/s00228-016-2129-7 27637913 PMC5203813

[B17] Schjøtt J. Reppe L. A. Roland P. D. Westergren T. (2012). A question-answer pair (QAP) database integrated with websites to answer complex questions submitted to the Regional Medicines Information and Pharmacovigilance Centres in Norway (RELIS): a descriptive study. BMJ Open 2, e000642 10.1136/bmjopen-2011-000642 PMC3307033 22422916

[B18] Schjøtt J. Schjøtt P. Assmus J. (2020). Analysis of consensus among drug interaction databases with regard to combinations of psychotropics. Basic Clin. Pharmacol. Toxicol. 126 (2), 126–132. 10.1111/bcpt.13312 31468698

[B19] Schoretsanitis G. de Leon J. Eap C. B. Kane J. M. Michael Paulzen M. (2019). Clinically significant drug-drug interactions with agents for attention-deficit/hyperactivity disorder. CNS Drugs 33 (12), 1201–1222. 10.1007/s40263-019-00683-7 31776871

[B20] Shah P. J. Morton M. J. S. (2013). Adults with attention-deficit hyperactivity disorder - diagnosis or normality? Br. J. Psychiatry 203 (5), 317–319. 10.1192/bjp.bp.113.126474 24187063

[B21] Sikirica V. Fridman M. Bruno A. Hodgkins P. Erder M. H. (2013). Concomitant pharmacotherapy of psychotropic medications in EU children and adolescents with attention-deficit/hyperactivity disorder. Drugs R 13 (4), 271–280. 10.1007/s40268-013-0034-4 PMC3851698 24271555

[B22] Stockley’s Interaction Checker (2020). MedicinesComplete®. Royal Pharmaceutical Society. Available at: http://www.medicinescomplete.com (Accessed September 15, 2020).

[B23] Thomas R. Sanders S. Doust J. Beller E. Glasziou P. (2015). Prevalence of attention-deficit/hyperactivity disorder: a systematic review and meta-analysis. Pediatrics 135 (4), 994–1001. 10.1542/peds.2014-3482 25733754

[B24] Ventola C. L. (2018). Big data and pharmacovigilance: data mining for adverse drug events and interactions. P T 43 (6), 340–351. 29896033 PMC5969211

[B25] Volkow N. D. Swanson J. M. (2013). Adult attention deficit–hyperactivity disorder. N. Engl. J. Med. 369 (20), 1935–1944. 10.1056/NEJMcp1212625 24224626 PMC4827421

[B26] WHO (2016). ATC/DDD index 2016. Available at: https://www.whocc.no/atc_ddd_index/ (Accessed September 18, 2020).

[B27] Willcutt E. G. (2012). The prevalence of DSM-IV attention-deficit/hyperactivity disorder: a meta-analytic review. Neurotherapeutics 9 (3), 490–499. 10.1007/s13311-012-0135-8 22976615 PMC3441936

[B28] Winterstein A. G. Soria-Saucedo R. Gerhard T. Correll C. U. Olfson M. (2017). Differential risk of increasing psychotropic polypharmacy use in children diagnosed with ADHD as preschoolers. J. Clin. Psychiatr. 78 (7), e744–e781. 10.4088/JCP.16m10884 28686819

[B29] Wu B. Bruns E. J. Tai M. H. Lee B. R. Raghavan R. dosReis S. (2018). Psychotropic polypharmacy among youths with serious emotional and behavioral disorders receiving coordinated care services. Psychiatr. Serv. 69 (6), 716–722. 10.1176/appi.ps.201700357 29540121

